# Concomitant Surgical Ablation Using a Novel Bipolar Radiofrequency Clamp: Outcomes from the TRAC-AF Registry

**DOI:** 10.3390/jcm14238360

**Published:** 2025-11-25

**Authors:** Christian Shults, Armin Kiankhooy, Shaf B. Holden, Hetal D. Patel, Frans van Wagenberg, Gansevoort H. Dunnington, Andres Samayoa, Theodore Wright, Andrew J. Sherman, Eric Sievers, Aaron Kime, Jeffrey Newman, Andrew Barksdale, Joshua N. Baugh, Yasir Abu-Omar, Gregory Rushing, Marc Gerdisch

**Affiliations:** 1 MedStar Washington Hospital Center, Washington, DC 20010, USA; 2 Cedars-Sinai Medical Center, Los Angeles, CA 90048, USA; 3 Huntsville Cardiothoracic Surgeons, Huntsville, AL 35801, USA; 4 Jackson-Madison County General Hospital, Jackson, TN 38305, USA; 5 Adventist-Health Saint Helena, Saint Helena, CA 94574, USA; 6 BayCare Health Systems, Tampa, FL 33759, USA; 7 Tenet Health Care, Delray Beach, FL 33484, USA; 8 Franciscan Health Indianapolis, Indianapolis, IN 46237, USA; 9 AtriCure, Inc., Mason, OH 45040, USA; 10 University Hospitals Cleveland Medical Center, Cleveland, OH 44106, USA

**Keywords:** surgical ablation, atrial fibrillation, left atrial appendage, cardiac surgery

## Abstract

**Background/Objectives**: Surgical ablation for atrial fibrillation (AF) during cardiac surgery decreases perioperative morbidity and mortality and improves long-term patient outcomes. Because of these benefits, it has been designated a Class I surgical society recommendation. As surgical ablation techniques have evolved, so too have ablation tools such as radiofrequency and cryothermal devices. In this study, we evaluated real-world evidence (RWE) of concomitant surgical ablation featuring an epicardial left atrial encircling lesion created by a novel bipolar radiofrequency clamp. **Methods**: Thirteen centers in the observational Tracking Results of Ablation to Combat AF (TRAC-AF) Registry (NCT05111015) contributed data used in this analysis. Included patients had AF and received the epicardial encircling lesion during cardiac surgery. Additional ablation and follow-up were per institutional standard of care. Freedom from AF/atrial tachycardia (AT)/atrial flutter (AFL) and survival were evaluated using the Kaplan–Meier method. Safety was evaluated within 30 days of the procedure. **Results**: Among 327 patients, 70% were male with a median age of 69 years. Sixty-nine percent had paroxysmal AF. Median left atrial diameter was 4.1 cm, and CHA_2_DS_2_-VASc score was 3. Isolated coronary artery bypass graft and aortic valve surgery were performed in 51% and 11% of patients, respectively. One- and two-year survival rates were 95.3% (95% CI, 91.7–97.3%) and 88.1% (95% CI, 81.5–92.5%). Through 12- and 24-months freedom from AF/AT/AFL was 87.4% (95% CI, 81.3–91.6%) and 79.9% (95% CI 72.0–85.8%). Mortality within 30 days of the index procedure was 1.5%. No serious adverse events were related to the epicardial cardiac ablation procedure or device. **Conclusions**: RWE from the TRAC-AF Registry demonstrates surgical ablation including an epicardial left atrial encircling lesion made by a novel bipolar RF clamp was safe and resulted in favorable long-term rhythm outcomes.

## 1. Introduction

Atrial fibrillation (AF) results in an increased risk of mortality, including sudden death. AF also leads to embolic stroke, transient ischemic attack (TIA), and worsening heart failure [1,2,3,4,5]. Most AF-related strokes originate in the left atrial appendage (LAA) [6]. The global prevalence of AF has grown markedly in recent years from 33.5 million people in 2010 to now affecting more than 59 million people worldwide, due to increased life expectancy. The risk of developing AF is associated with advanced age, with the highest incidence of AF occurring among Caucasian men living in western countries [7]. By 2030 it is estimated 12 million people will have AF in the United States, up from an estimated 8 million people currently living with AF [8,9].

Atrial fibrillation can be treated with surgical ablation at the time of cardiac surgery to restore normal sinus rhythm, which can increase left ventricular function, decrease perioperative morbidity and mortality, and improve long-term survival [10,11,12,13]. In addition, surgical LAA occlusion in patients with AF undergoing cardiac surgery has demonstrated a 33% reduction in thromboembolic events, compared to leaving the LAA intact [14]. Taken together, these benefits are reflected by Class I recommendations for concomitant surgical ablation and LAA exclusion during non-emergent cardiac surgery procedures per the 2023 Society of Thoracic Surgeons (STS) guidelines [15].

The most effective surgical ablation approach (including LAA closure) is the Cox-maze procedure, which is considered the gold standard [16]. It is a bilateral open-heart procedure intended to abolish the critical pathways responsible for AF by creating contiguous, transmural lesions with surgical incisions, and radiofrequency (RF) and cryothermal devices [17]. These devices have evolved in parallel with the variety of surgical ablation approaches developed to create critical lesions of the Cox-maze procedure, specifically the left atrial posterior wall and PV isolation.

Since late 2022, a novel bipolar RF clamp that encircles the left atrial posterior wall and PVs has been used clinically [18]. The encircling lesion minimizes aortic cross-clamp time by not requiring a left atriotomy to create this lesion [19,20]. Published clinical outcomes from the use of this clamp and approach are still emerging [19,20,21,22,23]. The aim of this study is to evaluate real-world perioperative safety and follow-up rhythm outcomes for cardiac surgery patients undergoing concomitant surgical ablation including an epicardial left atrial encircling lesion with the novel bipolar RF clamp.

## 2. Patients and Methods

### 2.1. Study Design

The Tracking Results of Ablation to Combat AF (TRAC-AF) Registry (NCT05111015) is a retrospective and prospective, multicenter observational registry established to collect real-world safety and effectiveness data on patients who undergo surgical or hybrid cardiac ablation and LAA management. The registry collects data per institutional standard of care. For this analysis, all patients with pre-operative AF at participating centers who underwent an epicardial left atrial encircling lesion during open cardiac surgery using the novel Isolator Synergy EnCompass^®^ clamp (AtriCure, Inc., Mason, OH, USA) were included.

### 2.2. Data Collection

Patients with pre-operative AF who underwent open cardiac surgery and were treated with concomitant surgical ablation Between 2022 and 2025 were included, with the last data pull performed on 8 July 2025. Patients enrolled prior to 11 March 2024, were part of Generation 1 of the TRAC-AF Registry database and thereafter using Generation 2 of the database. Data were collected by designated research staff at each participating center using standardized electronic case report forms (eCRFs) and entered into a secure online electronic database hosted by iMEDNET^™^ (Minnetonka, MN, USA).

Study personnel responsible for completing eCRFs underwent database training and were provided with a standardized abstraction guide to ensure uniform and standardized data collection. Data collection included baseline patient characteristics, demographics, comorbidities, cardiac surgery types and concomitant procedure types, left and right atrial lesion sets, LAA management, rhythm monitoring, mortality, medications and adverse events. Follow-up was performed per institutional standard of care. Device or procedure-related adverse events were required to be reported by study sites.

### 2.3. Statistical Methods

Rhythm outcomes were defined as freedom from AF, atrial tachycardia (AT), and atrial flutter (AFL) following a 90-day blanking period, through 12- and 24-months post procedure. Failure events for freedom from AF/AT/AFL were defined as >30 s recurrence of AF/AT/AFL, or a predominant rhythm status of AF/AT/AFL, or a repeat catheter ablation or cardioversion after the 90-day blanking period. In AF-only outcomes, repeat catheter ablation and/or a cardioversion after the 90-day blanking period were not counted as a failure. Mortality through 30 days and through two years was also analyzed. AF outcomes and long-term survival were estimated using the Kaplan–Meier method with a 95% confidence level. Patients were censored at date of last rhythm monitoring assessment (for arrhythmia Kaplan–Meier analyses) or date of last follow-up (for survival).

Safety outcomes included the incidence of serious procedure- and device-related adverse events experienced within 30 days of the index cardiac procedure. Additional outcomes of interest were incidence of stroke, and pacemaker implantation within 30 days. Site determination regarding relationship of adverse events to the device and/or procedure was only available in Generation 2 of the TRAC-AF Registry database. Descriptive statistics were reported using mean, standard deviation, median, minimum and maximum for continuous variables, and as percent (count/total) for categorical variables. Missing data were not imputed, thus only available data were assessed. SAS version 9.4 was used for the analysis.

### 2.4. Surgical Procedures and Techniques

The EnCompass clamp was used in all open concomitant surgical ablations in this study. This bipolar RF clamp comes in two jaw lengths, a standard size with an electrode length of 83 mm and the long size with an electrode length of 106 mm. Choice of the clamp size was at the discretion of the physician in this study. A red rubber magnetic guide is used with the device to pass through the transverse and oblique sinuses and aid in pulling the clamp into position for ablation. In effect, the EnCompass device can encircle the left atrial posterior wall and PV ostia in a single application. A detailed review of the surgical procedure and positioning of the Encompass device during open cardiac surgery is available in Torregrossa et al., 2024 [20].

**Figure 1:**
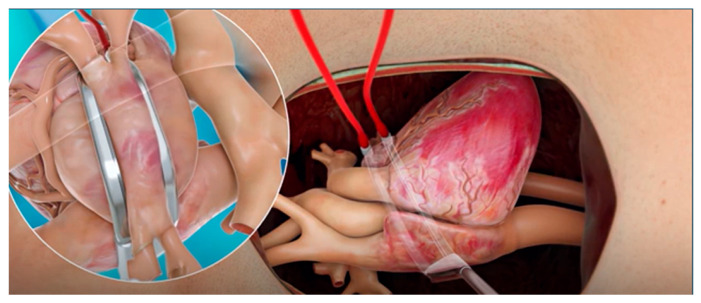
Surgical ablation illustration of the left atrial posterior wall encircling lesion using the bipolar RF clamp.

All patients included in this analysis had received at minimum a left atrial encircling lesion with the bipolar RF clamp, see Figure 1. Additional left and right sided lesions, as well as LAA management were performed at the surgeon’s discretion and per institutional standard of care.

## 3. Results

### 3.1. Baseline Characteristics

Data from 327 patients enrolled in the TRAC-AF Registry (54 from Generation 1 and 273 from Generation 2), across 13 centers in the US were included in this analysis (Supplemental Figure S1). Median age was 69 years (IQR, 63–75 years) and 70% of patients were male. Most patients had paroxysmal AF at baseline (69%), followed by persistent AF (14%), long-standing persistent AF (5%), early persistent AF (3%) and 9% had an unknown AF classification at baseline (Table 1).

The median left atrial diameter was 4.1 cm (IQR, 3.4–4.7) with a left ventricle ejection fraction (LVEF) of 55% (IQR, 40.0–60.0). Median BMI was 29.8 (IQR, 26.3–34.5) kg/m^2^, with a median CHA_2_DS_2_-VASc score of 3 (IQR, 2–4) and a HAS-BLED score of 2 (IQR, 1–3). Comorbidities and risk factors included hypertension in 86% of patients, coronary heart disease (50%), heart failure (40%), diabetes mellitus (36%), smoking or history of tobacco use (37%), sleep apnea (21%), previous stroke/TIA (12%) and chronic obstructive pulmonary disease (11%). At baseline, 19% of patients had a prior cardioversion and 9% a catheter ablation. Five patients had greater than one prior catheter ablation (Table 1).

### 3.2. Cardiac Surgeries and Ablations Performed

Index cardiac surgeries included isolated coronary artery bypass graft (CABG) in 51%, isolated aortic valve (AVR/r) in 11%, isolated mitral valve (MVR/r) in 9%, CABG with AVR/r in 8%, CABG with MVR/r in 5%, AVR/r and MVR/r in 2% of patients. Other cardiac procedures performed included aortic aneurysm (7%) and tricuspid valve (TVR/r) (6%) (Table 2).

Surgical ablation lesion sets were predominantly left atrial posterior wall box lesions alone, performed in 56% of patients. Additional left-sided lesions were performed in 19% of patients, right-sided in 8%, and bi-atrial lesions in 18% of patients. One patient had the left side lesions of the Cox-maze procedure; however, no patients had a full bilateral Cox-maze. The most common additional left-sided lesion performed was the LAA to the left superior pulmonary vein, in 21% of patients. The most common additional right atrial lesions were the superior vena cava (23%) and inferior vena cava (23%). Among patients who received LAA exclusion, AtriClip^®^ (AtriCure, Inc., Mason, OH, USA) was used in 98.1% (Table 3).

### 3.3. Survival

**Figure 2:**
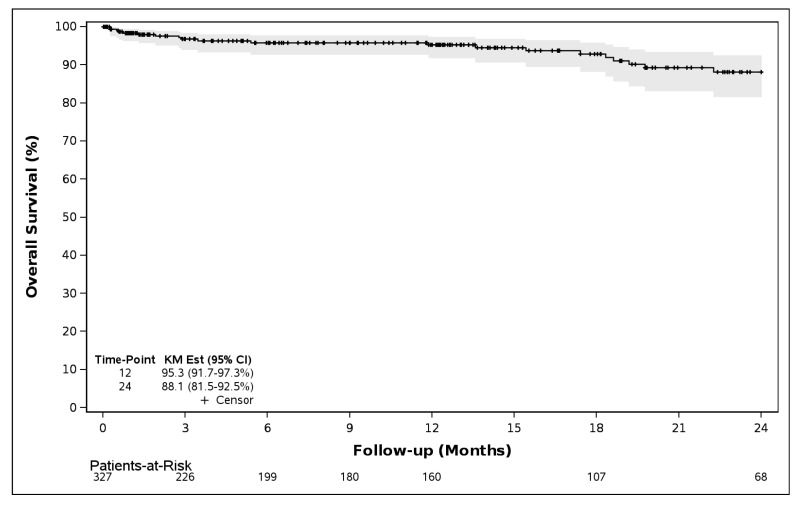
Survival through 12 months and 24 months (N = 327).

Survival through one year was 95.3% (95% CI, 91.7–97.3%) and through two years was 88.1% (95% CI, 81.5–92.5%), see Figure 2.

### 3.4. Follow up and Rhythm Outcomes

The median follow-up duration was 12.8 months (range 0–39 months), among the whole cohort of 327 patients. Evaluable rhythm data (including failure for reintervention) after the 90-day blanking period were available from 198 patients. Patient characteristics and demographics at baseline, including only those patients with evaluable rhythm data (n = 198) are available in Supplemental Table S1. During follow-up, rhythm monitoring was performed by ECG in 61.8% of patients, ECG and non-ECG monitoring in 14.7% of patients, and non-ECG monitoring in 14.1% of patients. Among patients with non-paroxysmal AF at baseline, monitoring was by ECG in 62.1%, both ECG and non-ECG monitoring in 10.3%, and non-ECG in 20.7%.

**Figure 3:**
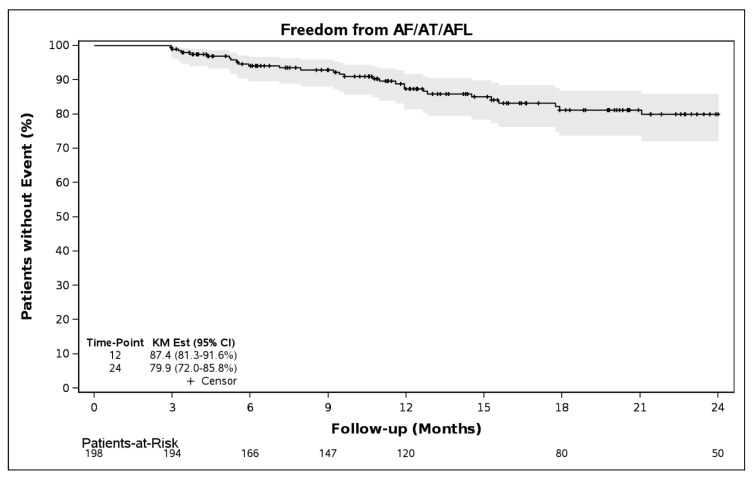
Freedom from atrial fibrillation, (AF), atrial tachycardia (AT), and atrial flutter (AFL), following a 90-day blanking period (N = 198).

**Figure 4:**
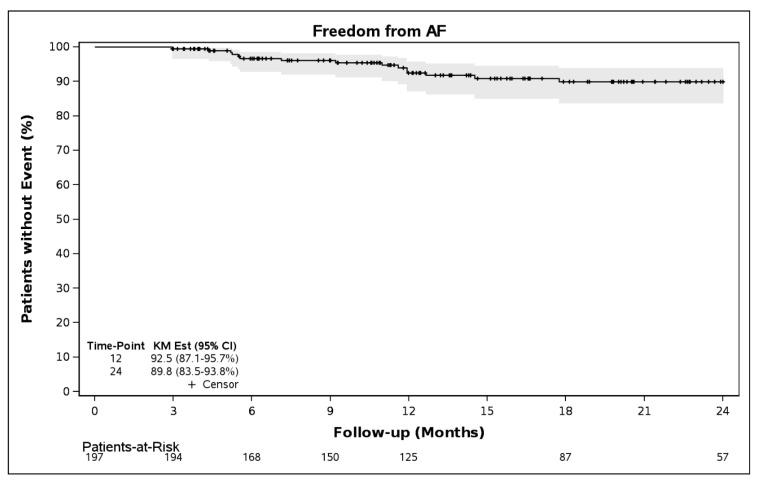
Freedom from atrial fibrillation (AF) following a 90-day blanking period (N = 197).

Freedom from AF/AT/AFL was 87.4% (95% CI, 81.3–91.6%) and 79.9% (95% CI 72.0–85.8%), through 12 and 24 months, respectively (Figure 3). Freedom from AF alone through 12 months was 92.5% (95% CI, 87.1–95.7%) and through 24 months was 89.8% (95% CI, 83.5–93.8%, Figure 4). Class I and III antiarrhythmic drug (AAD) data were available on 126 patients, who also had evaluable rhythm data. Through 12 months, 85.7% (108/126) of patients were off AADs and free from AF/AT/AFL.

In patients with non-paroxysmal AF at baseline, freedom from AF/AT/AFL was 80.5% (95% CI, 58.5–91.6%) through 12 months. Freedom from AF alone, was the same in this group at 80.5%. In patients with paroxysmal AF at baseline, freedom from AF/AT/AFL was 89.4% (95% CI, 82.4–93.7%) through 12 months and freedom from AF alone was 96.6% (95% CI, 91.2–98.7%) through 12 months. Rhythm outcomes across these groups are displayed in Table 4. In patients treated with a left atrial box lesion alone, without any additional lesions, freedom from AF/AT/AFL was 88.2% (95% CI 80.1–93.1%) through 12 months. Freedom from AF alone through 12 months, was 91.2% (95%CI 83.6–95.3%, Supplemental Figures S2 and S3).

**Figure 5:**
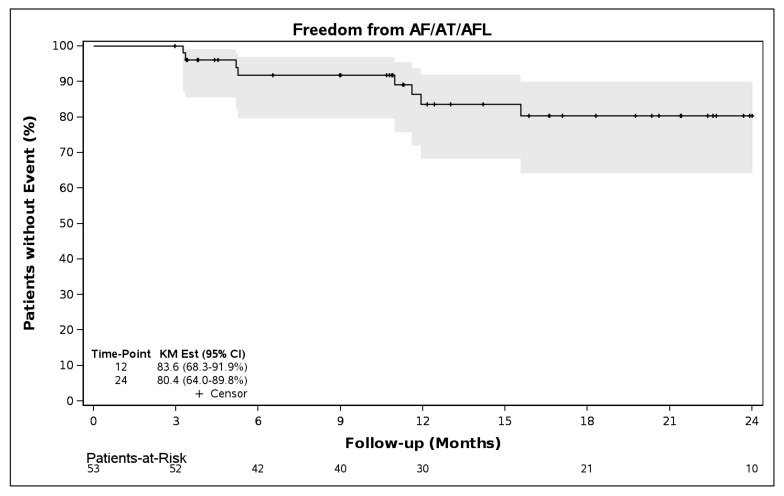
Freedom from AF/AFL/AT following a 90-day blanking period in patients with non-ECG monitoring (N = 53).

**Figure 6:**
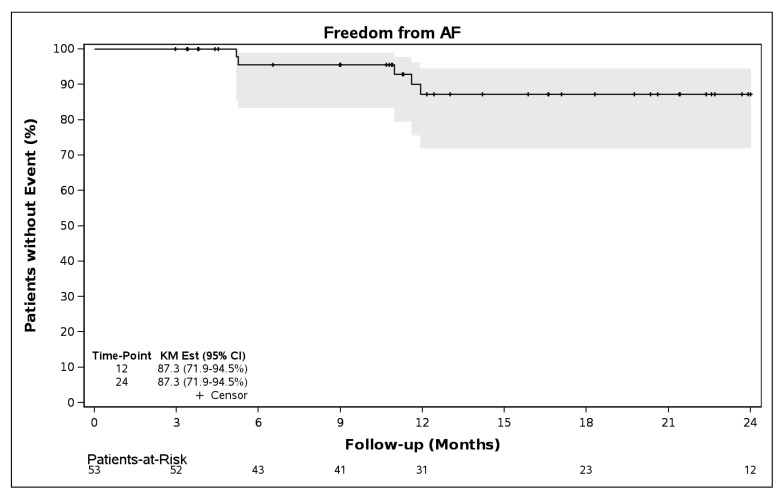
Freedom from AF following a 90-day blanking period in patients with non-ECG monitoring (N = 53).

A subanalysis of patients who had at least one continuous (non-ECG) monitoring assessment (n = 53) showed freedom from AF/AFL/AT was 83.6% (95% CI 68.3–91.9%) though 12 months and 80.4% (95% 64.0–89.8%) through 24 months (Figure 5). Freedom from AF was 87.3% (71.9–94.5%) through both 12 and 24 months in this subgroup (Figure 6).

### 3.5. Safety

Mortality within 30 days of the index procedure was 1.5% (5/327). The permanent pacemaker (PPM) implantation rate was 6.1% (20/327) within 30 days. Neurologic adverse events were observed in 1/327 (0.3%) patients, and the ischemic stroke was resolved without sequalae. No serious adverse events were related to the epicardial cardiac ablation procedure or device.

## 4. Discussion

The EnCompass clamp enables encircling of the posterior wall and PVs with just one application, simplifying the process of surgical ablation and minimizing aortic cross-clamp time [19]. Recent experiences with this device demonstrated no intraoperative complications and early efficacy [21,22,23]. Makati et al., reported outcomes of 12 patients with non-paroxysmal AF who underwent concomitant ablation with the EnCompass device and cryoablation plus LAA exclusion with AtriClip [22]. No complications were observed. Additionally, Rushing et al. reported a series of 35 patients who underwent coronary revascularization and ablation with the EnCompass device and cryoablation plus LAA exclusion during cardiac surgery without the need for atriotomy [23]. There were no operative deaths or intraoperative complications, and no patients required a PPM. One patient experienced a post-operative stroke. Freedom from AF/AT was 96% through both 12 and 24 months in 22 and 17 patients, respectively. A multicenter study on the non-atriotomy box lesion in 83 CABG patients reported 95% freedom from AF with mean follow-up of 12.5 months [21].

The current study builds on these findings. Approximately 68% of patients underwent a non-atriotomy index procedure (CABG ± AVR/r or aortic aneurysm) and most patients (56%) were treated with a left atrial box lesion alone. In addition, 98.2% of patients in this study also underwent concomitant LAA exclusion. The 30-day mortality rate of 1.5% in this study is comparable to the rate of 1.7% in the CABG population from the STS database [24]. The PPM rate of 6.1% within 30 days is less than 7.6% observed in the STS data. Furthermore, the rate of neurologic adverse events in this study of 0.3% is below the 1.8% reported in STS [24,25].

Rhythm outcomes among patients treated with concomitant left atrial surgical ablation vary widely, with efficacy rates reported for surgical ablation between 44 and 94% through 1 year, and may be impacted by age, AF type, comorbidities, index cardiac procedure, and several other factors [26,27]. CABG patients experience a greater freedom from AF of 80–89% [15,28]. This study’s rates of 87.4% efficacy through 12 months and 79.9% through 24 months are comparable to these findings. For paroxysmal AF, a left atrial box lesion with LAA management has demonstrated an 80% efficacy rate in non-atriotomy procedures [29]. Patients treated with a left atrial box lesion alone in this study demonstrated freedom from AF/AFL/AT of 88.2% through 12 months and the rate remained high, 82.6% through 24 months, indicating durability.

Limitations to the current study include the observational and retrospective/prospective registry design. Furthermore, data were collected per institutional standard of care, with no mandated follow-up or method for rhythm monitoring. Follow up and rhythm data were available in 61% of patients (198/327), which may indicate these patients have not yet completed follow-up or there is no documented follow-up available. TRAC-AF Registry data reflect institutional standards of care, with most rhythm monitoring data collected using ECG; however, patients who received ECGs during an office visit had multiple office visits, which included rhythm and symptom monitoring to ascertain arrhythmia status. The predominance of solely ECG as the rhythm monitoring method underscores the need for better utilization of continuous ambulatory monitoring after surgical ablation for AF. It has been shown that the rigor of the rhythm monitoring strategy can have a significant impact on arrhythmia detection [30]. Thus, to obtain a true picture of AF ablation success and rhythm outcomes, there must be a call to action to more robustly track rhythm outcomes post-ablation.

Despite these limitations, more than 39 surgeons treating patients with surgical ablation at 13 centers across the United States participated in this large multicenter study. A robust definition for rhythm failure events, capturing any 30 s recurrence, a predominant rhythm status change, or a repeat catheter ablation/cardioversion was employed. Data entry was standardized, and data quality checks were performed for missing data and outliers. These data reflect the real-world experience and outcomes of patients treated with the EnCompass clamp.

## 5. Conclusions

We report multicenter real-world registry data on use of the EnCompass clamp for an epicardial left atrial encircling lesion in patients with AF undergoing concomitant surgical ablation. This study demonstrates real-world evidence supporting safety, effectiveness, and durability of this ablation approach.

## Figures and Tables

**Table 1:** Patient characteristics and demographics at baseline.

Characteristic	Total Treated (N = 327)
Age, years	
Mean ± SD (n)	68.1 ± 8.8 (327)
Median (Q1, Q3)	69.0 (63.0, 75.0)
Sex	
Female	29.7% (97/327)
Male	70.3% (230/327)
Body mass index, kg/m^2^	
Mean ± SD (n)	30.9 ± 7.0 (326)
Median (Q1, Q3)	29.8 (26.3, 34.5)
Atrial fibrillation type	
Early Persistent	2.8% (9/327)
Long-Standing Persistent	5.2% (17/327)
Paroxysmal	69.4% (227/327)
Persistent	14.1% (46/327)
Unknown	8.6% (28/327)
CHA_2_DS_2-_VASc score	
Mean ± SD (n)	3.3 ± 1.5 (327)
Median (Q1, Q3)	3.0 (2.0, 4.0)
HAS-BLED score	
Mean ± SD (n)	2.2 ± 1.3 (327)
Median (Q1, Q3)	2.0 (1.0, 3.0)
Medical History	
Previous stroke/TIA	11.6% (38/327)
Diabetes	35.8% (117/327)
Heart failure	39.8% (130/327)
Chronic obstructive pulmonary disease	10.7% (35/327)
Coronary heart disease	50.2% (164/327)
Sleep apnea	20.8% (68/327)
Smoking/tobacco use history	37.0% (121/327)
Hypertension	85.9% (281/327)
Previous cardioversions	18.7% (61/327)
Previous catheter ablations	8.6% (28/327)
1	6.1% (20/327)
2	0.9% (3/327)
3+	0.6% (2/327)
Unknown	0.9% (3/327)
Left Ventricular Ejection Fraction (%)	
Mean ± SD (n)	50.6 ± 13.5 (238) *
Median (Q1, Q3)	55.0 (40.0, 60.0)
Left Atrial Diameter (cm)	
Mean ± SD (n)	4.1 ± 1.0 (160) *
Median (Q1, Q3)	4.1 (3.4, 4.7)

* Data not available on left ventricular ejection fraction and left atrial diameter for all patients.

**Table 2:** Index cardiac procedures performed, categorized per STS database [24] (N = 327).

Cardiac Procedure Types	% (n/N)
Isolated CABG	50.5% (165/327)
Isolated Aortic Valve replacement/repair (AVR/r)	11.0% (36/327)
Isolated Mitral Valve replacement/repair (MVR/r)	8.9% (29/327)
AVR/r + CABG	8.0% (26/327)
MVR/r + CABG	5.2% (17/327)
AVR/r + MVR/r	1.5% (5/327)
Aortic Aneurysm *	6.7% (22/327)
Tricuspid Valve replacement/repair (TVR/r)	5.5% (18/327)
Other	2.8% (9/327)

* Aortic Aneurysm includes ascending aortic replacement with or without aortic root replacement, and/or with or without aortic arch replacement.

**Table 3:** Concomitant cardiac ablation procedures and left atrial appendage (LAA) management. (N = 327).

Concomitant Procedures	% (n/N)
Box lesion *, no additional lesions	56.0% (183/327)
Box lesion * + Additional left-sided lesions	18.7% (61/327)
Box lesion * + Additional right-sided lesions	7.6% (25/327)
Box lesion * + Both left- and right-sided lesions	17.7% (58/327)
Box lesion * + Full left-sided maze	0.3% (1/327)
Concomitant LAA exclusion	98.2% (320/326)
AtriClip	98.1% (314/320)
Surgical Stapling	0.9% (3/320)
Surgical Amputation	0.3% (1/320)
Surgical Ligation	0.3% (1/320)
Penditure™	0.3% (1/320)

* Left atrial posterior wall ablation performed using the bipolar RF clamp.

**Table 4:** Rhythm outcomes through 12 months with 95% confidence interval.

	All Patients (N = 198)	Paroxysmal AF (N = 141)	Non-Paroxysmal AF (N = 31)
Freedom from AF/AT/AFL:	87.4% (81.3–91.6)	89.4% (82.4–93.7)	80.5% (58.5–91.6)
Freedom from AF:	92.5% (87.1–95.7)	96.6% (91.2–98.7)	80.5% (58.5–91.6)

## Data Availability

The datasets presented in this article are not readily available as data are part of an ongoing study.

## Supplementary Materials

The following supporting information can be downloaded at: https://www.mdpi.com/article/10.3390/jcm14238360/s1, Figure S1: Flow diagram describing patient population and exclusions; Figure S2: Patients treated with left atrial box lesion alone. Freedom from atrial fibrillation, (AF), atrial tachycardia (AT), and atrial flutter (AFL), following a 90-day blanking period (N = 126); Figure S3: Patients treated with left atrial box lesion alone. Freedom from atrial fibrillation (AF) following a 90-day blanking period (N = 126); Table S1: Patient characteristics and demographics at baseline, including only patients with evaluable rhythm data following a 90-day blanking period (N = 198).

## Abbreviations

The following abbreviations are used in this manuscript:

AF	Atrial fibrillation
AFL	Atrial flutter
AT	Atrial tachycardia
AVR/r	Aortic valve repair/replacement
CABG	Coronary artery bypass graft
LAA	Left atrial appendage
MVR/r	Mitral valve repair/replacement
PPM	Permanent pacemaker
RF	Radiofrequency
SAE	Serious adverse event
TRAC-AF	Tracking Results of Ablation to Combat AF
TVR/r	Tricuspid valve repair/replacement
