# Supplementary material for: Concomitant Surgical Ablation Using a Novel Bipolar Radiofrequency Clamp: Outcomes from the TRAC-AF Registry
Source: J Clin Med. 2025 Nov 25;14(23):8360. doi: 10.3390/jcm14238360 (PMC12692788; doi:10.3390/jcm14238360)
Supplement: Supplementary file 1 [file jcm-14-08360-s001.zip › jcm-3970973-supplementary.pdf]

Supplementary Materials

Figure S1. Flow diagram describing patient population and exclusions.

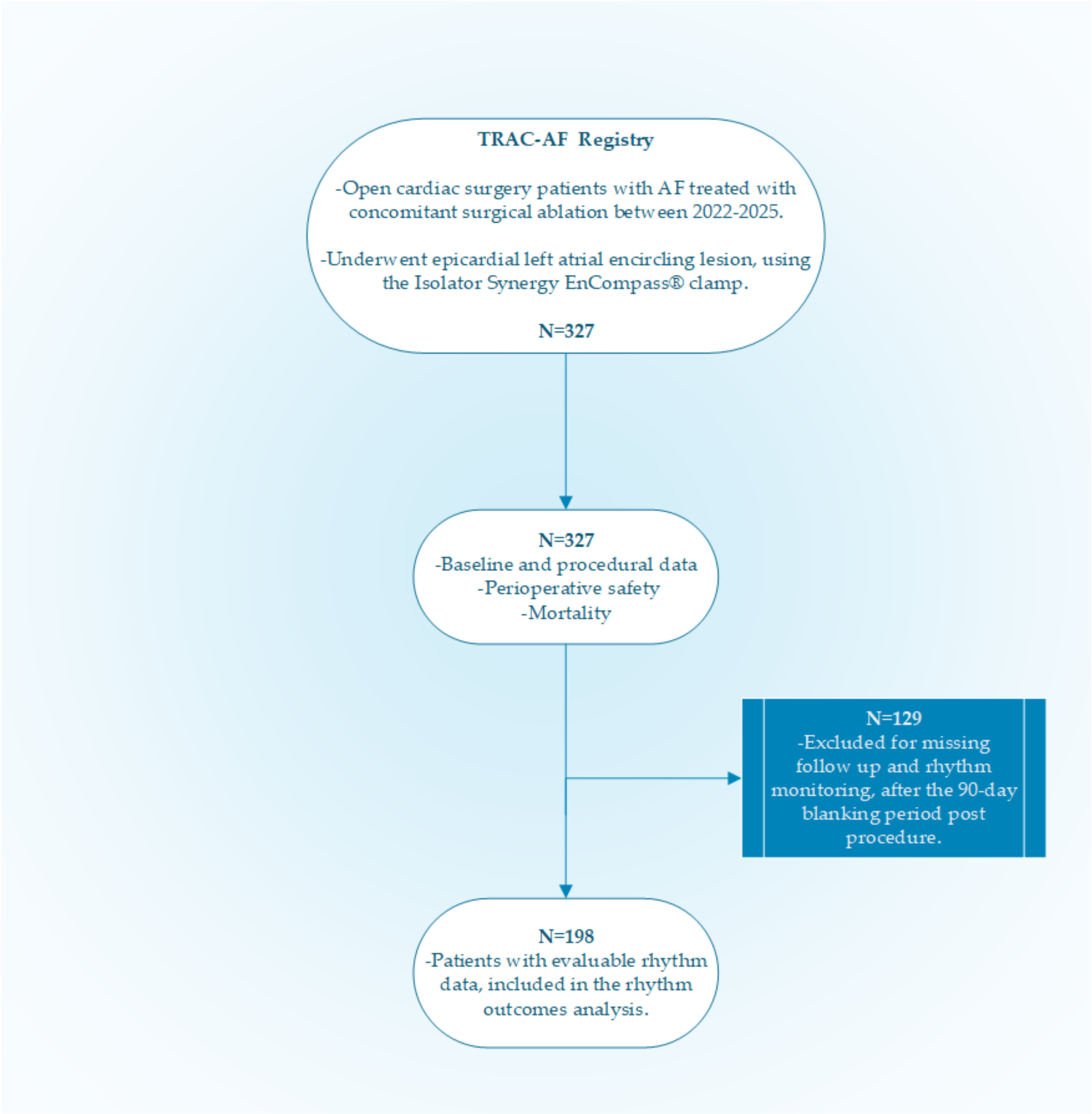

Figure S2. Patients treated with left atrial box lesion alone. Freedom from atrial fibrillation, (AF), atrial tachycardia (AT), and atrial flutter (AFL), following a 90-day blanking period (N=126).

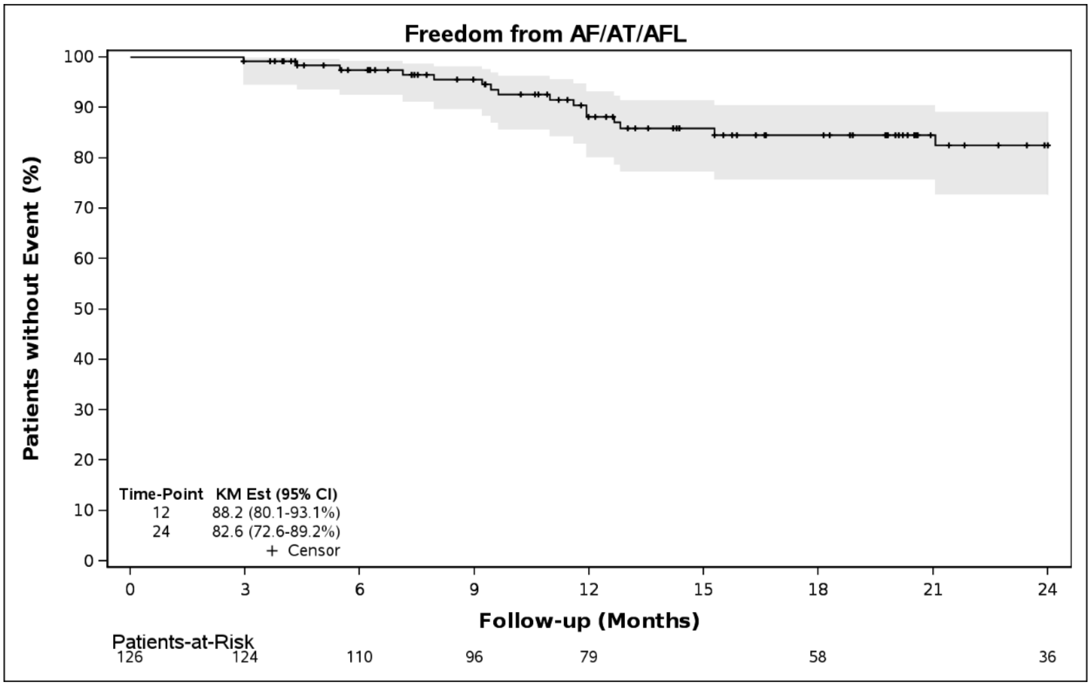

Figure S3. Patients treated with left atrial box lesion alone. Freedom from atrial fibrillation (AF) following a 90-day blanking period (N=126).

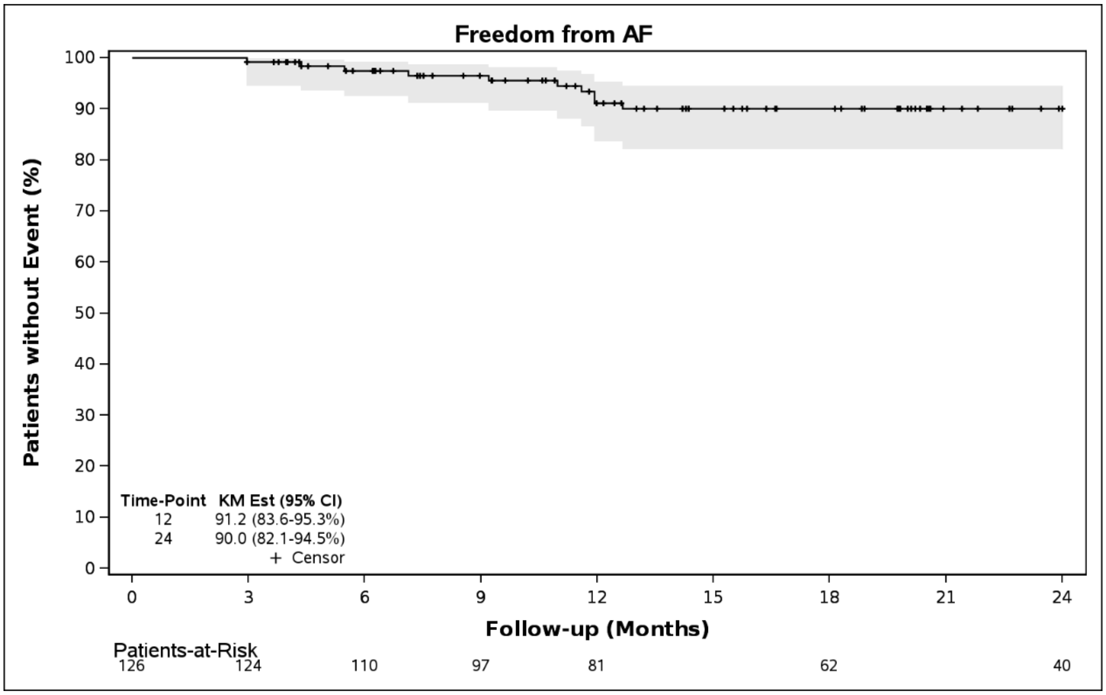

Table S1. Patient characteristics and demographics at baseline, including only patients with evaluable rhythm data following a 90-day blanking period (N=198).

**Characteristic**	Total Treated (N=198)
Age, years	
Mean ± SD (n)	67.7 ± 8.6 (198)
Median (Q1, Q3)	69.0 (63.0, 74.0)
Sex	
Female	28.8% (57/198)
Male	71.2% (141/198)
Body mass index, kg/m^2	
Mean ± SD (n)	31.2 ± 7.3 (198)
Median (Q1, Q3)	30.1 (26.5, 34.6)
Atrial fibrillation type	
Early Persistent	1.0% (2/198)
Long-Standing Persistent	3.5% (7/198)
Paroxysmal	71.2% (141/198)
Persistent	11.1% (22/198)
Unknown	13.1% (26/198)
CHA_2DS_2VASc score	
Mean ± SD (n)	3.2 ± 1.4 (198)
Median (Q1, Q3)	3.0 (2.0, 4.0)
HAS-BLED score	
Mean ± SD (n)	2.2 ± 1.3 (198)
Median (Q1, Q3)	2.0 (1.0, 3.0)
Medical History	
Previous stroke/TIA	10.6% (21/198)
Diabetes	38.4% (76/198)
Heart failure	37.9% (75/198)
Chronic obstructive pulmonary disease	13.1% (26/198)
Coronary heart disease	48.0% (95/198)

**Characteristic**	Total Treated (N=198)
Sleep apnea	22.7% (45/198)
Smoking/tobacco use history	41.4% (82/198)
Hypertension	89.9% (178/198)
Previous cardioversions	15.2% (30/198)
Previous catheter ablations	7.1% (14/198)
1	4.0% (8/198)
2	1.0% (2/198)
3+	0.5% (1/198)
Unknown	1.5% (3/198)
Left Ventricular Ejection Fraction (%)	
Mean ± SD (n)	51.1 ± 13.0 (163)
Median (Q1, Q3)	55.0 (43.0, 60.0)
Left Atrial Diameter (cm)	
Mean ± SD (n)	4.2 ± 0.9 (97)
Median (Q1, Q3)	4.3 (3.6, 4.7)
